# Target product profiles: leprosy diagnostics

**DOI:** 10.2471/BLT.23.290881

**Published:** 2024-02-22

**Authors:** Petra Kukkaro, Sundeep Chaitanya Vedithi, David J Blok, Wim H van Brakel, Annemieke Geluk, Aparna Srikantam, David Scollard, Linda B Adams, Mathias Duck, Sunil Anand, Andie Tucker, Israel Cruz, VRR Pemmaraju, Daniel Argaw Dagne, Kingsley Asiedu, Christopher Hanna

**Affiliations:** a Novartis Pharma AG, Basel, Switzerland.; b Department of Biochemistry, University of Cambridge, Cambridge, England.; c Department of Public Health, Erasmus University Medical Center, Rotterdam, Kingdom of the Netherlands.; d Until No Leprosy Remains, Amsterdam, Kingdom of the Netherlands.; e Department of Infectious Diseases, Leiden University Medical Center, Leiden, Kingdom of the Netherlands.; f Clinical and Laboratory Research Division, Blue Peter Public Health and Research Centre-LEPRA Society, Hyderabad, India.; g National Hansen’s Disease Program, Baton Rouge, United States of America (USA).; h The Leprosy Mission International, Brentford, England.; i American Leprosy Missions, Hyderabad, India.; j The Task Force for Global Health, Inc, Decatur, USA.; k National School of Public Health, Instituto de Salud Carlos III, Madrid, Spain.; l Global Leprosy Programme, WHO Regional Office for South-East Asia, New Delhi, India.; m Department of Control of Neglected Tropical Diseases, World Health Organization, Geneva, Switzerland.; n Global Project Partners, Oakland, USA.

## Abstract

The World Health Organization (WHO) aims to reduce new leprosy cases by 70% by 2030, necessitating advancements in leprosy diagnostics. Here we discuss the development of two WHO's target product profiles for such diagnostics. These profiles define criteria for product use, design, performance, configuration and distribution, with a focus on accessibility and affordability. The first target product profile outlines requirements for tests to confirm diagnosis of leprosy in individuals with clinical signs and symptoms, to guide multidrug treatment initiation. The second target product profile outlines requirements for tests to detect *Mycobacterium leprae* or *M. lepromatosis* infection among asymptomatic contacts of leprosy patients, aiding prophylactic interventions and prevention. Statistical modelling was used to assess sensitivity and specificity requirements for these diagnostic tests. The paper highlights challenges in achieving high specificity, given the varying endemicity of *M. leprae,* and identifying target analytes with robust performance across leprosy phenotypes. We conclude that diagnostics with appropriate product design and performance characteristics are crucial for early detection and preventive intervention, advocating for the transition from leprosy management to prevention.

## Résumé

L'Organisation mondiale de la Santé (OMS) vise à réduire le nombre de nouveaux cas de lèpre de 70% d'ici 2030, ce qui nécessite un meilleur diagnostic de la maladie. Dans le présent document, nous évoquons le développement de deux profils de produit cible établis par l'OMS à cette fin. Ces profils définissent des critères en matière d'utilisation, de conception, de performances, de configuration et de distribution du produit, en accordant une attention particulière à l'accessibilité et à l'abordabilité. Le premier profil de produit cible décrit les exigences pour les tests servant à confirmer le diagnostic de la lèpre chez les individus qui présentent des signes cliniques et des symptômes, afin d'orienter l'instauration d'un traitement à base de plusieurs médicaments. Le second profil de produit cible décrit les exigences pour les tests servant à détecter une infection à *Mycobacterium leprae* ou *M. lepromatosis* parmi les contacts asymptomatiques de patients lépreux, ce qui contribue à l'adoption de mesures prophylactiques et à la prévention. Nous avons eu recours à une modélisation statistique pour évaluer les exigences de sensibilité et de spécificité de ces tests diagnostiques. Cet article met en évidence les obstacles à l'atteinte d'un niveau élevé de spécificité en raison de l'endémicité variable de *M. leprae,* et à l'identification d'analytes cibles offrant de bons résultats chez les phénotypes lépreux. Nous concluons qu'un diagnostic reposant sur des caractéristiques de performance et de conception appropriées est essentiel pour détecter rapidement la maladie et intervenir en amont, et nous plaidons pour une prévention plutôt qu'une gestion de la lèpre.

## Resumen

La Organización Mundial de la Salud (OMS) pretende reducir los nuevos casos de lepra en un 70% para 2030, lo que requiere avances en el diagnóstico de la lepra. Aquí se analiza el desarrollo de dos perfiles de productos objetivo de la OMS para este tipo de diagnósticos. Estos perfiles definen los criterios de uso, diseño, rendimiento, configuración y distribución de los productos, centrándose en su accesibilidad y asequibilidad. El primer perfil de producto objetivo describe los requisitos de las pruebas para confirmar el diagnóstico de la lepra en personas con signos y síntomas clínicos, con el fin de orientar el inicio del tratamiento con múltiples fármacos. El segundo perfil de producto objetivo describe los requisitos de las pruebas para detectar la infección por *Mycobacterium leprae* o *M. lepromatosis* entre los contactos asintomáticos de los pacientes con lepra, para facilitar las intervenciones profilácticas y la prevención. Se utilizaron modelos estadísticos para evaluar los requisitos de sensibilidad y especificidad de estas pruebas diagnósticas. El artículo destaca las dificultades para lograr una alta especificidad, dada la diferente endemicidad de *M. leprae*, y para identificar analitos diana con un rendimiento sólido en todos los fenotipos de lepra. Concluimos que los diagnósticos con un diseño de producto y unas características de rendimiento adecuados son fundamentales para la detección precoz y la intervención preventiva, lo que favorece la transición del manejo de la lepra a la prevención.

## ملخص

تهدف منظمة الصحة العالمية (WHO) إلى خفض حالات الإصابة الجديدة بالجذام بنسبة %70 بحلول عام 2030، مما يستلزم إحراز خطوات للتقدم في تشخيص مرض الجذام. نحن نناقش هنا تطوير اثنين من ملفات تعريف المنتجات المستهدفة لدى منظمة الصحة العالمية لمثل هذه التشخيصات. تحدد هذه الملفات التعريفية معايير استخدام المنتج، وتصميمه، وأداؤه، وتكوينه، وتوزيعه، مع التركيز على إمكانية الحصول عليه، والقدرة على تحمل تكاليفه. يحدد الملف التعريفي الأول للمنتج المستهدف متطلبات الاختبارات لتأكيد تشخيص الإصابة بالجذام لدى الأفراد الذين يعانون من علامات وأعراض إكلينيكية، وذلك لإدارة بدء العلاج بأدوية متعددة. ويحدد ملف تعريف المنتج المستهدف الثاني متطلبات اختبارات الكشف عن عدوى المتفطرة الجذامية أو متفطرة الجذام بين مخالطي مرضى الجذام، ولا تظهر عليهم الأعراض، مما يساعد في التدخلات الوقائية والعلاجية. تم اللجوء لوضع نموذج إحصائية لتقييم متطلبات الحساسية والخصوصية لهذه الاختبارات التشخيصية. تركز هذه الورقة على التحديات التي تواجه تحقيق خصوصية عالية، في ظل الاستشراء المتباين للمتفطرة الجذامية، وتحديد التحليلات المستهدفة ذات الأداء القوي عبر النماذج الظاهرية للجذام. وننتهي إلى أن التشخيص باستخدام التصميم المناسب للمنتج وخصائص الأداء، هو أمر حيوي للكشف المبكر والتدخل الوقائي، والدعوة إلى الانتقال من إدارة مرض الجذام إلى الوقاية.

## 摘要

世界卫生组织 (WHO) 的目标是到 2030 年将新发麻风病病例减少 70%,这需要改进麻风病的诊断方法。在本文,我们将讨论世卫组织针对此类诊断产品制定的两个目标产品简介。这些产品简介定义了产品使用、设计、性能、配置和分发的标准,重点关注了产品的可及性和可负担性。第一份目标产品简介概述了对有临床体征和症状的个体进行检测以确诊麻风病的要求,以在启动多药治疗时提供指导。第二份目标产品简介概述了对麻风病患者的无症状接触者进行检测以查明*麻风分枝杆菌*或*弥散型麻风分枝杆菌*感染情况的要求,这有助于采取预防性干预和预防措施。统计模型用于评估这些诊断试验的敏感性和特异性要求。本文考虑到不同地方*麻风分枝杆菌*的流行状况,强调了实现高特异性和识别在各种麻风表型中表现稳定的靶分析物方面所面临的挑战。我们得出结论,使用具有适当产品设计和性能特征的诊断产品对于早期检测和采取预防性干预措施至关重要,倡导从麻风病的诊治转向麻风病的预防。

## Резюме

Всемирная организация здравоохранения (ВОЗ) ставит перед собой задачу сократить число новых случаев лепры на 70% к 2030 году, что требует совершенствования методов диагностики заболевания. В этой статье представлена информация о разработке двух профилей целевых продуктов ВОЗ для таких диагностических средств. На основе этих профилей определяются критерии использования, дизайна, эффективности, конфигурации и распространения продукции с акцентом на физическую и экономическую доступность. В первом профиле целевого продукта изложены требования к тестам для подтверждения диагноза лепры у лиц с клиническими проявлениями и симптомами с целью определения начала лечения несколькими препаратами. Второй профиль целевого продукта определяет требования к тестам для выявления инфекции *Mycobacterium leprae* или *M. lepromatosis* среди бессимптомных контактов больных лепрой, что способствует проведению профилактических мероприятий и предотвращению распространения заболевания. Для оценки требований к чувствительности и специфичности этих диагностических тестов использовался метод статистического моделирования. В статье освещаются проблемы достижения высокой специфичности с учетом различной эндемичности *M. leprae*, а также выявления целевых аналитов, позволяющих надежно определять заболевание при различных фенотипах лепры. Авторы пришли к выводу, что диагностическая продукция с соответствующим дизайном и эксплуатационными характеристиками имеет решающее значение для выявления заболевания на ранних стадиях и проведения профилактических мероприятий, способствуя переходу от лечения лепры к ее профилактике.

## Introduction

Leprosy, a neglected tropical disease, is caused by *Mycobacterium leprae* or less often by *M. lepromatosis.*^1^ The disease is a chronic, moderately infectious condition affecting mostly skin, peripheral nerves, mucosa of upper respiratory tract and eyes.^2^^,^^3^ Approximately 200 000 new cases are reported annually from nearly 120 countries.^4^ In 2021, the World Health Organization (WHO) launched the *Towards zero leprosy: global leprosy (‎Hansen’s disease)‎ strategy 2021–2030*, aiming for a global reduction of 70% in new leprosy cases by 2030.^5^ Continued investment in leprosy diagnostics is crucial if we are to achieve the proposed targets.

Leprosy is an important public health problem due to its potential for causing lasting physical impairments and adverse socioeconomic consequences if left undiagnosed.^6^^,^^7^ *M. leprae* is moderately contagious and infections can become chronic. Infected contacts can remain asymptomatic for up to 20 years,^8^^–^^10^ and indirect evidence suggests that these individuals with subclinical infection could transmit *M. leprae* to close contacts.^10^^–^^13^ Therefore, conducting regular contact tracing and testing, and administering prophylactic treatment is crucial to interrupt transmission cycles.^11^ Despite multidrug treatment availability and global advancements in leprosy treatment, delayed diagnosis remains a substantial concern as it can lead to grade 2 disabilities (ulcers, contractures, foot drop, lagophthalmos, and muscle wasting).^14^^,^^15^ The global reduction in leprosy cases,^16^ along with less active engagement from health-care professionals in managing the disease, has led to a decline in clinical public health expertise in diagnosing leprosy, further causing delays in diagnosis.^15^^,^^17^^,^^18^

Currently, leprosy diagnosis primarily relies on defined clinical criteria.^19^ Microscopic or laboratory-based diagnosis using acid-fast bacilli identification in a slit-skin smear or skin biopsy is used in numerous leprosy programmes and tertiary care settings.^19^ Additionally, various point-of-care tests and laboratory assays have been developed to detect *M. leprae* infection directly or indirectly.^20^^–^^22^ These include enzyme-linked immunosorbent assays and lateral flow assays for detection of immunoglobulins and polymerase chain reaction for pathogen detection.^23^^,^^24^ Both immunodiagnostics and molecular assays are sensitive enough to diagnose multibacillary leprosy^23^ as well as some paucibacillary cases.^25^ Although direct diagnosis of paucibacillary leprosy is challenging, in vitro stimulation followed by detection of immunity against *M. leprae* antigens, increases diagnostic potential.^26^^,^^27^

Variability in leprosy presentation, patient type and diagnostic targets complicate accurate testing.^28^^,^^29^ Furthermore, limited awareness about leprosy among health-care workers poses a diagnostic challenge.^30^ Hence, diagnostic tests that support rapid contact tracing and screening are essential for efficient and comprehensive leprosy control programmes. Easy-to-use diagnostic tests are therefore needed to help reduce delays.

In efforts to achieve better performance, some tests lean on complex instrumentation and expertise that limit their field use, especially in low-resource settings. Additionally, tests requiring invasive sampling are challenging to deploy in a field setting.^31^ These problems underscore the current need to develop diagnostic tests designed for settings where they are most needed.^32^

To facilitate early prophylactic interventions to disrupt the chain of leprosy transmission, the Global Partnership for Zero Leprosy, under WHO's diagnostic technical advisory group guidance,^33^ developed two target product profiles for high-priority leprosy diagnostics. These profiles ensure that the diagnostic products not only meet the necessary performance criteria but also consider the specificities of the intended health-care context and the patient demographic. The first target product profile covers confirmatory diagnostic tests for individuals presenting with clinical manifestations indicative of leprosy, with the goal of initiating multidrug therapy. The second target product profile covers diagnostic assays for the detection of *M. leprae* infection in asymptomatic households or familial contacts of individuals with confirmed clinical leprosy. 

## Methods

### Development process

To create target product profiles and guide product developers, the Global Partnership for Zero Leprosy formed a leprosy-focused diagnostic expert working group to assist WHO’s diagnostic technical advisory group’s skin neglected tropical disease subgroup. The working group assisted by clarifying unmet public health needs; determining whether existing available target product profiles or pipeline products are addressing current needs; defining the scope of needed new target product profiles; and serving as a scientific group to develop new target product profiles.

The Global Partnership for Zero Leprosy included leprosy experts working in laboratory, field research and clinical capacities, as well as community stakeholders who developed target product profiles. The group collaborated with the WHO diagnostic technical advisory group, WHO Technical Advisory Group on Leprosy Control, and consulted experts at the Bill & Melinda Gates Foundation. The original draft version criteria were chosen by the Global Partnership for Zero Leprosy’s diagnostic working group using methods such as landscape assessments, use case needs analyses and diagnostic performance modelling, all designed through an internal consultative process.

The Global Partnership for Zero Leprosy’s diagnostic working group reviewed the need for a leprosy diagnostic test using WHO reports, literature and outcomes of discussions with the experts. We created the first version of target product profile using the quality by design planning method,^34^^,^^35^ with performance characteristics based on statistical analysis and modelling by expert group members (online repository).^36^ Based on feedback from diagnostic technical advisory group members, we adapted the first version before publishing the document on the WHO website for public consultation for one month (30 November to 30 December 2021). In addition to the online public consultation, the WHO Technical Advisory Group on Leprosy Control also reviewed the first version. To finalize the target product profile, we addressed all comments received on the first version, and subsequently the chair of the diagnostic technical advisory group’s skin neglected tropical diseases subgroup and a WHO technical staff member reviewed the document.

## Target product profiles^9^^,^^10^

WHO finalized and disseminated final version of the target product profiles on 24 July 2023.^9^^,^^10^ Target product profile 1 describes a test to confirm leprosy in individuals presenting with clinical signs and symptoms (hereafter confirmatory test).^9^ Target product profile 2 describes a point-of-care test for the detection of analytes specific to *M*. *leprae* or host response to *M. leprae* to enable detection of subclinical *M. leprae* infections (hereafter test for subclinical infection).^10^

The WHO diagnostic target product profiles define minimal and ideal targets for each profile and organize them into five categories: (i) product use summary; (ii) design; (iii) performance; (iv) product configuration; and (v) product costs and distribution channels. Minimal refers to the lowest acceptable output for a characteristic for the test to be suitable for the intended use, and ideal reflects targets that may be harder to achieve but would accelerate access, adoption and clinical outcomes. 

### Product use summary

For a confirmatory test, the intended application is at minimum a laboratory-based assay for the qualitative and quantitative detection of biomarkers specific to *M. leprae* and, ideally, *M. lepromatosis*. This test should be able to confirm diagnosis of clinical leprosy in individuals exhibiting clinical manifestations. In contrast, the subclinical test delineates specifications for a point-of-care, rapid diagnostic tool aimed at identifying biomarkers pertinent to *M. leprae* or the host immune response to *M. leprae* or *M. lepromatosis.* Such a test should be applicable in contact tracing scenarios, and facilitate detection of asymptomatic *M. leprae* infections among contacts of leprosy patients. The ideal intended use for both profiles is deployment as point-of-care diagnostics.

A confirmatory test should require minimal infrastructure, characterized by a laboratory setting where technicians with less than one week of additional formal training can perform the assay. The ideal scenario for such a test is a point-of-care format executable in health-care settings without any laboratory infrastructure. The ideal intended user profile is health-care professionals, community health workers and volunteers; requiring only a one-day training, complemented by easy and accessible usage instructions. For tests for subclinical infection, the prerequisites for infrastructure, end-user capability and training are consistent with the ideal conditions described for confirmatory tests.

### Design

For confirmatory tests, at the minimal level, portability requisites for a laboratory-based assay stipulate that transport and portability conditions should not exceed those of standard laboratory apparatus. The test should be designed to use electricity supplied by main lines and laboratory-grade water resources (such as distilled water). Should instrumentation require periodic maintenance and calibration, it should be feasible within the recipient countries and not more than once per calendar year. Acceptable specimen types include capillary blood via fingerstick, venous blood, collected urine, nasal swabs, slit-skin smears and punch biopsies, with the latter permitting sub-millimetre tissue collection. Sample processing and transfer should be simple, necessitating a single holding tube with a 500 µL capacity and disposable transfer pipette for one-time use. The maximum sample volume should not surpass 100 µL. Confirmatory tests should aim to detect biomarkers uniquely associated with *M. leprae* and provide semiquantitative analysis of bacterial load or immune response. An instrument-based detection method should incorporate an external process control indicator. All necessary reagents and operational supplies must comply with the basic importation restrictions and ensure the safety of the operator.

In an ideal scenario, confirmatory tests should be done on a highly portable point-of-care device without specialized transport requirements. The device should be battery powered or otherwise not depend on mains power and availability of water, obviating the need for regular maintenance or calibration. Sample collection is confined to capillary blood via fingerstick, urine or nasal swabs, with straightforward processing and single-use pipette transfer. Ideally, requisite sample volume is less than 10 µL. The assay should quantitatively determine biomarkers specific to both *M. leprae* and *M. lepromatosis*, assessing bacterial load or immune status. Results should be discernible to the unaided eye, marked by stark contrast and clarity. The required provisions for quality control, necessary supplies and safety protocols mirror those at the minimal level.

For a test for subclinical infection, the prerequisites for portability, and power and water independence are consistent for both minimal and ideal characteristics. The test's portability should negate the need for specialized transportation, mains electricity and water supply. At the minimum level, any field-compatible equipment employed (e.g. sample incubator, reader) should require only basic maintenance or calibration, potentially facilitated through return to the manufacturer or execution of a standard procedure. In the ideal scenario, the reader should require neither maintenance nor calibration. Both minimal and ideal acceptable sample types include capillary blood, collected urine and nasal swabs, with venous blood and slit-skin smear included at the minimal level only. Sample volumes are confined to less than 100 µL for the minimal scenario and less than 10 µL for the ideal scenario. The minimal requirement is identification of biomarkers indicative of latent *M. leprae* infection, whereas in the ideal scenario the test also identifies *M. lepromatosis*. Both in minimal and ideal scenarios a qualitative output is favoured, with results clearly visible to the naked eye, and the test must have an internal process control indicator.

### Performance

For confirmatory tests, a minimal diagnostic assay should display a clinical sensitivity of ≥ 90% and a specificity of ≥ 99% for the detection of *M. leprae*. The assay should yield results within 4 hours, and these results must maintain their stability for at least 30 minutes post-analysis. Operational throughput should exceed 100 tests per technician per day. Assay stability should be ≥ 18 months when stored at temperatures ranging from 4 °C to 40 °C and at 75% relative humidity. The testing procedure should be limited to maximum 15 user steps, of which a maximum of five steps should be timed. 

An ideal confirmatory test would be capable of detecting both *M. leprae* and *M. lepromatosis*, maintaining a clinical sensitivity of ≥ 90% but with a specificity of ≥ 99.9%. A field-deployable version of the assay should deliver results in less than 30 minutes, with the stability of results extending to at least 24 hours. Operational throughput should surpass 10 tests per technician per hour. The stability criterion for the ideal test should extend to ≥ 24 months under the aforementioned temperature and humidity conditions. Conducting the analysis should be possible by performing maximum five steps, out of which no more than one should be timed.

For a test for subclinical infection, the minimal test should have a clinical sensitivity of ≥ 81% and a specificity of ≥ 99.5% for the detection of *M. leprae*. The time to results should be less than 2 hours, and these results should maintain their stability for at least 30 minutes post-analysis. The expected throughput is more than seven tests per tester per hour. The stability of these assays should be no less than 18 months within the temperature range of 4 °C to 40 °C and at maximum 75% relative humidity. The analysis should be maximum two timed steps and maximum eight user steps. 

An ideal test for subclinical infection would detect both *M. leprae* and *M. lepromatosis* with a clinical sensitivity of ≥ 94% and a specificity of ≥ 99.9%. The test should produce results in less than 30 minutes, with results remaining stable for at least 24 hours. The throughput should be more than 10 tests per tester per hour, with stability guaranteed for ≥ 24 months under the above specified storage conditions. The test should be designed for ease of use, with maximum one timed step and maximum five user steps. Both minimal and ideal tests should yield binary outcomes.

### Product configuration

A minimal confirmatory test must adhere to the relevant standards, such as ASTM International (ASTM) D4169–05 and international standard organization (ISO) 11607–1:2006, or their equivalents. Test components or consumables for laboratory use should be able to be stored and shipped at temperatures ranging from 0 °C to 4 °C. Cold storage is an acceptable condition for any laboratory-based assays. For laboratory-based tests, support should be available from the equipment manufacturer for problem-solving and use of the equipment.

All materials included in the assay should be universally compatible with standard laboratory biohazard waste management protocols. Labelling and instructions must comply with the pertinent CE Mark under In Vitro Diagnostic Regulation stipulations (or other recognized regulatory authorities, such as the United States Federal Drug Administration under 21 CFR 820), alongside guidelines set forth by the WHO prequalification processes.^37^

The ideal product configuration for confirmatory tests and both the minimal and ideal scenarios for tests for subclinical infection have the same requirements. These assays should be point-of-care tests that adhere to the specified ASTM and ISO standards or their accepted equivalents, eliminating the need for cold-chain transport. They should be storable at ambient temperatures ranging from 2 °C to 40 °C without requiring service interventions. The assays must not include any materials that are not compatible with standard biohazard waste disposal procedures in a laboratory environment. Packaging must consider daily throughput to minimize unnecessary waste. Finally, the labelling and usage instructions should align with those established for the minimal requirements for confirmatory tests.

### Product cost and channels

The minimal requirements for the cost of a confirmatory test are below 3 United States dollars (US$). Capital expenditure for the deployment of such tests should remain within a threshold of US$ 5000. The anticipated lead time for product availability should be less than eight weeks. The market introduction should be focused on countries with endemic leprosy, with regulatory prerequisites encompassing: (i) compliance with CE Mark under In Vitro Diagnostic Regulation or other relevant stringent regulatory authorities; (ii) export certifications from the country of manufacture; (iii) WHO prequalification, contingent upon necessity and relevance; and (iv) national registration in accordance with the regulatory demands of target countries.

The pricing for an ideal confirmatory test is set at below US$ 1, not accounting for additional expenses like logistics, storage and other operational costs related to national procurement for neglected tropical diseases programmes. Capital costs for these laboratory-based tests should not exceed US$ 5000 in a minimal scenario; however, ideally, given the point-of-care nature of the test, no capital investment would be required. The expected lead time for the product should be less than six weeks. The target markets and required registrations for launch should match those outlined for the minimal test.

For a test for subclinical infection, the financial and distribution criteria remain consistent with those for the ideal confirmatory test, with the stipulation that capital costs can go up to US$ 2000 for both the minimal and ideal versions. Minimally, the contact-tracing test is specifically designed for countries that are actively involved in leprosy contact tracing and post-exposure prophylaxis programmes.

### Comparative analysis 

**Fig. 1:**
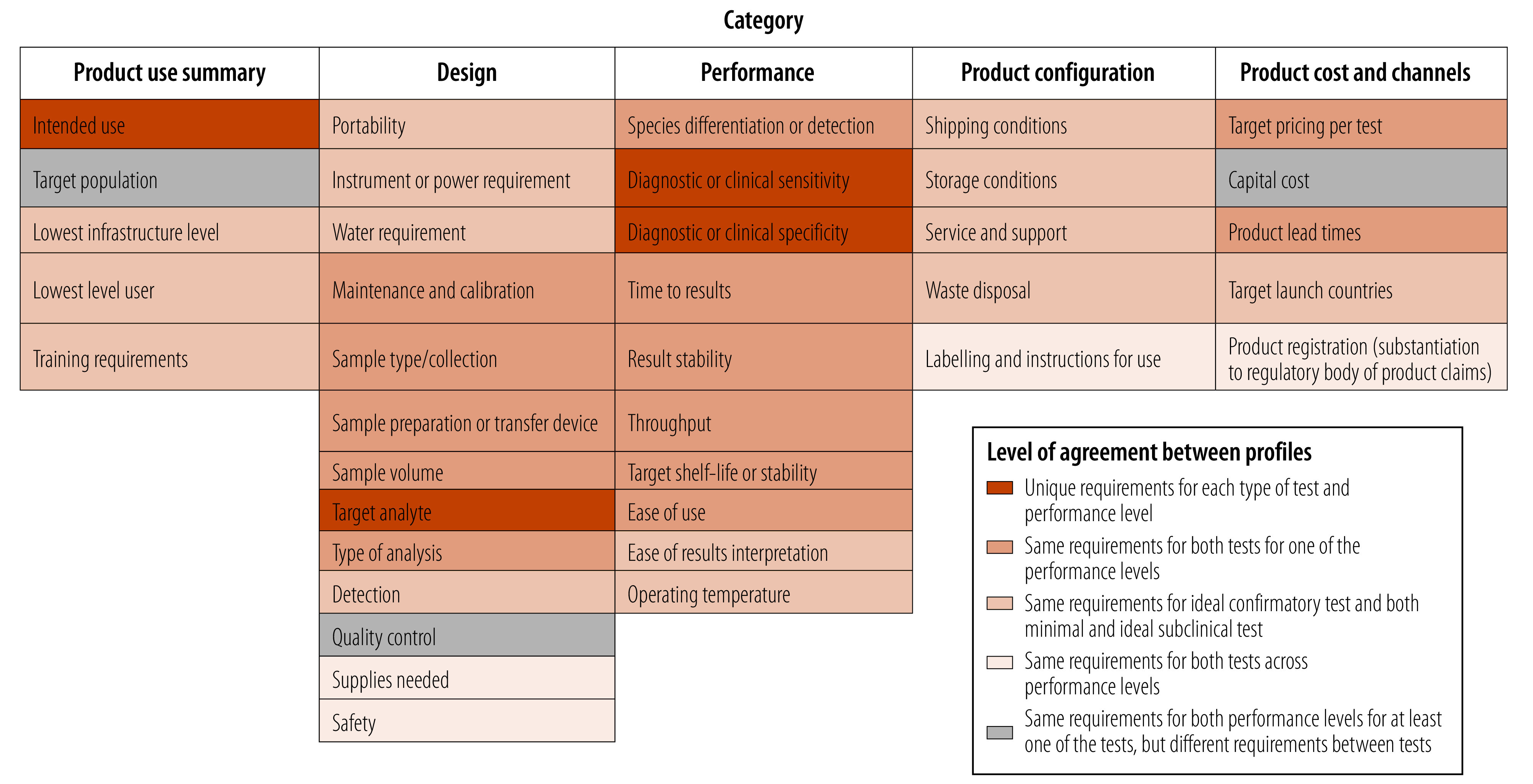
Comparative analysis matrix for target product profiles for leprosy diagnostics

Note: Target product profile 1 is a diagnostic test to confirm leprosy in individuals presenting with clinical signs and symptoms;^9^ and target product profile 2 is a diagnostic test to identify *Mycobacterium leprae* infection in asymptomatic household and family contacts of diagnosed leprosy patients.^10^

When comparing 38 requirements across the five categories for both minimal and ideal requirements, we found that 10.5% (4/38) of the requirements were identical for both profiles, regardless of whether we were looking at the minimal or ideal criteria. When comparing the ideal confirmatory test with both levels of test for subclinical infection, 36.8% (14/38) of the requirements were the same. Furthermore, 34.2% (13/38) of the requirements were alike for both types of tests when considering either the minimal or ideal scenario. Only 18.4% (7/38) of the requirements were different between the two types of tests (Fig. 1).

## Discussion

Here we have outlined the minimal and ideal requirements listed in the target product profiles for a confirmatory test and contact-tracing test for leprosy.^9^^,^^10^ Development of a new test complying with the requirements could improve testing outcomes, avoiding complications such as unethical treatment, emotional and physical effects, social exclusion and higher costs.^38^

Three high-risk factors must be considered to ensure the successful development of a diagnostic test for leprosy, as highlighted for both target product profiles. First, the diagnostic tests aim to identify biomarkers indicative of active infection with *M. leprae* and *M. lepromatosis*. However, *M. leprae* may remain dormant for years and reactivate under certain immune conditions, complicating the identification of recent infections. Despite the challenges and time required to qualify and validate new markers, it is crucial to advance test development using the currently available analytes to prevent delays in development. Second, due to the similar clinical presentations of other mycobacterial infections (like *M. tuberculosis*) and skin disorders prevalent in the same areas as leprosy is prevalent, the tests must differentiate between these conditions. The development of multiplex assays, which can detect multiple diseases simultaneously, could streamline patient care and diagnostics. Third, even in highly endemic areas, the prevalence of leprosy cases is so low that it poses unique diagnostic challenges; the specificity requirements are high;^39^ and the tests must be highly specific to avoid false positives and unnecessary treatment. While test developers may find these performance targets challenging, they are crucial to achieve and maintain low rates of both false positives and false negatives, especially as diseases approach elimination. Perfect accuracy is rarely possible with a single test. However, using multiple tests together, either serial testing or parallel testing, can improve the overall accuracy. Serial testing uses a sequence of more precise tests to confirm a diagnosis, while parallel testing checks for several disease indicators at once to enhance the detection process.^39^

The target product profile outlines that one of the minimal requirements for a confirmatory test is high specificity for all types of leprosy, including manifestations with low bacilli level. While such specificity can be difficult to achieve, some studies demonstrated adequate sensitivity across paucibacillary and multibacillary leprosy.^40^^–^^43^ To ensure adequate performance across leprosy manifestations, clinical validation studies should be set up at multiple sites worldwide to demonstrate that acceptance criteria are met for all types of leprosy. 

As leprosy incidence decreases, and confirmation of diagnosis happens in environments that can support moderately complex evaluations, laboratory-based tests can satisfy the minimal requirements for a confirmatory diagnosis of leprosy. Given the strict clinical sensitivity and specificity requirements, laboratory-based tests might be more suitable than point-of-care tests to meet these performance criteria. On the contrary, to perform WHO-recommended contact tracing for individuals diagnosed with *M. leprae* infection, a test that can be implemented at the point-of-care is essential to ensure usefulness in field settings. In all cases, ensuring immediate availability of appropriate medical interventions following the detection of leprosy or *M. leprae* infection is crucial for ethical reasons. 

Currently, WHO only recommends use of single-dose rifampicin for post-exposure-prophylaxsis.^11^ However, in household contacts of newly diagnosed leprosy cases, a single dose of rifampicin may not suffice, as the observed risk reduction for developing leprosy is only 50%–60% and this protection lasts for merely two years after administration.^44^^,^^45^ Thus, additional tools to detect *M. leprae* infection, together with improved post-exposure prophylaxis, are desirable. Close collaboration, coordination and alignment are required with teams working on other post-exposure-prophylaxis regimens, to ensure concurrent availability of these regimens along with the appropriate diagnostic tools. Two trials are currently ongoing: one in Bangladesh, Brazil, India and Nepal, and another in the Comoros.^46^^,^^47^ In these trials, field teams closely collaborate with researchers developing and evaluating immunodiagnostic and molecular tests for monitoring the direct and longitudinal effects and efficacy of various forms of post-exposure prophylaxis on development of leprosy.^46^^,^^47^ Clinical validation studies of both diagnostics and treatment interventions may depend on and benefit from each other.

A factor not covered in this research is the impact of stigma associated with leprosy, leading to discrimination against affected individuals and their families, which can hinder timely diagnosis and treatment. Long-standing stigmas associated with leprosy necessitate diagnostic approaches that also consider social factors such as privacy and discretion, similar to contact-tracing efforts undertaken in human immunodeficiency virus testing. 

In conclusion, investing in diagnostics for both disease and infection is critical to significantly reduce new cases of leprosy worldwide. WHO target product profiles for leprosy diagnostics can help guide development of appropriate tools. The goal is to not only manage leprosy but also to prevent it, thereby reducing its global burden.
